# A CRISPR-Cas9 system protecting *E.* *coli* against acquisition of antibiotic resistance genes

**DOI:** 10.1038/s41598-025-85334-2

**Published:** 2025-01-09

**Authors:** Danna Lee, Petra Muir, Sara Lundberg, August Lundholm, Linus Sandegren, Sanna Koskiniemi

**Affiliations:** 1 Department of Cell and Molecular Biology, Uppsala University, Uppsala, Sweden; 2 Department of Medical Biochemistry and Microbiology, Uppsala University, Uppsala, Sweden

**Keywords:** Antibiotics, Bacterial synthetic biology, Bacterial genes, Applied microbiology, Bacteria, Bacteriology

## Abstract

Antimicrobial resistance (AMR) is an increasing problem worldwide, and new treatment options for bacterial infections are direly needed. Engineered probiotics show strong potential in treating or preventing bacterial infections. However, one concern with the use of live bacteria is the risk of the bacteria acquiring genes encoding for AMR or virulence factors through horizontal gene transfer (HGT), and the transformation of the probiotic into a superbug. Therefore, we developed an engineered CRISPR-Cas9 system that protects bacteria from horizontal gene transfer. We synthesized a CRISPR locus targeting eight AMR genes and cloned this with the Cas9 and transacting tracrRNA on a medium copy plasmid. We next evaluated the efficiency of the system to block HGT through transformation, transduction, and conjugation. Our results show that expression of the CRISPR-Cas9 system successfully protects *E. coli* MG1655 from acquiring the targeted resistance genes by transformation or transduction with 2–3 logs of protection depending on the system for transfer and the target gene. Furthermore, we show that the system blocks conjugation of a set of clinical plasmids, and that the system is also able to protect the probiotic bacterium *E. coli* Nissle 1917 from acquiring AMR genes.

**Supplementary Information:**

The online version contains supplementary material available at 10.1038/s41598-025-85334-2.

## Introduction

Antimicrobial resistant (AMR) bacterial infections pose a significant and growing global threat, with an estimated burden of over 1.2 million deaths worldwide in 2019 directly caused by resistant bacteria, and millions more indirectly^1^. AMR arising *de novo* from spontaneous mutations typically provides resistance by reduced antibiotic uptake, increased antibiotic efflux through upregulation of endogenous pump systems, or structural alteration of the target molecule^2^. Additionally, common resistance mechanisms acquired through horizontal gene transfer (HGT) include antibiotic-specific efflux pumps, alternate metabolic pathways, enzymatic modifications of antibiotic targets, or direct enzymatic inactivation of antibiotics^3^. The intense use of antibiotics world-wide has promoted the mobilization and horizontal transfer of such resistance genes to many pathogenic bacterial species^4^, making HGT the most important factor driving AMR^5^.

Horizontally transferred genes constitute a large part of most bacterial genomes, and the transfer of genes encoding for AMR or virulence factors is important for the emergence of new pathogenic strains^6^. Genes spread amongst microbial communities via different modes of HGT, namely: (i) transformation (direct uptake of DNA from the extracellular environment into bacterial cells)^7^, (ii) transduction (mediated by phage packaging of host genetic material)^8^, and (iii) conjugation (direct transfer of plasmids between bacterial cells via conjugative machinery)^9^.

With the emergence of multidrug-resistant pathogenic strains, new approaches are needed to combat this rising global health crisis. A promising alternative to traditional antibiotics is the use of probiotic bacteria. Probiotics, defined as live microorganisms that confer health benefits upon ingestion^10^, possess the potential to combat pathogenic bacteria. Extensive research has explored the use of probiotics in treating various gastrointestinal diseases. Among others, irritable bowel syndrome, antibiotic-associated diarrhea, and antibiotics-induced *Clostridioides difficile* disease have shown positive effects when treated with probiotics^11^. However, the precise mechanisms of how different probiotics protect the host from pathogenic bacteria are not entirely understood (reviewed in^12^). Filling this knowledge void will be important for increasing the efficacy of probiotics, but requires significant efforts from the research community. In the meantime, genetically engineered probiotics allow a more defined way to treat specific diseases, with reported applications ranging from cancer and wound healing to conditions like phenylketonuria, inflammatory bowel disease, and infection (reviewed in^13^). One of the most frequent starting strains for such approaches is the commercially available probiotic strain *E. coli* Nissle 1917^13^, which is excellent at colonizing the human gut^14^ and has been associated with many beneficial traits even without genetic modification^15^. Numerous attempts to genetically engineer *E. coli* Nissle to prevent diseases ranging from hangover (patent nr: 20240175039) to cancer (patent nr: 11123380) exist (reviewed in^16^). But a critical concern in probiotic use, including the use of engineered *E. coli* Nissle, is the potential acquisition of virulence factors and AMR genes from pathogenic strains through HGT within the human gut^17^. Likewise, the ability of probiotic strains to transfer their genetic traits to other gut bacteria warrants careful consideration. Consequently, it is important to develop means to safeguard probiotic strains against HGT while also implementing measures to biocontain potential probiotic strains.

One of the many naturally occurring bacterial defence systems against HGT is CRISPR-Cas (*c*lustered *r*egularly *i*nterspaced *s*hort *p*alindromic *r*epeats-CRISPR-associated proteins)^18^. The CRISPR array consists of short repeated sequences separated by similarly short unique sequences called spacers, often matching exogenic sequences such as phages and plasmids^19–21^. The system also encodes Cas proteins to perform immunity functions. Many types of CRISPR systems (types I-VI) have been identified in various bacterial and archaeal hosts^22^. Here, we will focus on the Type II CRISPR system, which is one of the best characterized and more easily engineered then for example the type-I-E system found naturally in *E. coli*, where multiple proteins make up the endonuclease^23–25^. The type II system consists of Cas1-2, the endonuclease Cas9, the CRISPR array encoding guide RNAs, and the trans-activating tracrRNA^24^. CRISPR-Cas immunity is carried out in three main stages: adaptation, expression, and interference.

During adaptation, Cas1 and Cas2 incorporate new spacers from incoming phage or plasmid DNA into the CRISPR array. This process can be either naïve, where spacers are acquired from a sequence not yet targeted by a spacer, requiring no interference machinery^26^, or primed, where the interference machinery guides adaptation to a sequence that fully or partially matches a crRNA^27–30^. Next, the CRISPR array and the remaining *cas* genes are expressed, and the pre-CRISPR RNA is processed into RNA guides by Cas9 and the tracrRNA. Each guide RNA contains a 20nt-long guide sequence and palindromic RNA, which serves as the handle for the Cas9 nuclease to bind to. For interference, the 20nt guide then directs the Cas9-RNA complex to the correct DNA sequence by Watson-Crick base pairing^31^, resulting in cleavage and elimination of the invading DNA^18^. To protect the cell from self-destruction, the targeted sequence, or protospacer, is always associated with a protospacer adjacent motif (PAM), which for the CRISPR-Cas system derived from *Streptococcus pyogenes* (used here) is 5’-NGG. The PAM is essential for interference. Structural analyses show that interaction between Cas9 and the NGG motif allows melting of the double stranded DNA, providing access for the guide RNA to base pair with the DNA target^32^. This motif is absent in the CRISPR locus, thereby protecting the DNA sequence from cleavage upon CRISPR expression^25^. Thus, when foreign DNA enters a bacterium, through whatever mechanism, where the CRISPR RNA and Cas9 are expressed, RNA guided cleavage of the incoming DNA protects the cell against HGT. Investigation of CRISPR spacers found in nature, suggests that CRISPR systems provide protection against HGT of plasmids as well as phage^33,34^. An excellent review on CRISPR systems can be found in^35^.

Several studies have explored the idea of using CRISPR-Cas systems to empower genetically engineered probiotics, by e.g. delivering CRISPR-Cas systems to specific pathogenic or drug-resistant bacteria to kill them or clear them of their antimicrobial resistance^36–43^. In a study by Kim et al.^44^, a CRISPR-Cas9 system targeting a conserved sequence in TEM- and SHV-type extended-spectrum β-lactamases (ESBLs) could be delivered into ESBL-producing *E. coli* and restored antibiotic susceptibility^44^. Similarly, Hao et al.^40^ used CRISPR-Cas9 to cure carbapenemase genes and plasmids in *Enterobacteriaceae*, resulting in efficient resensitizing to carbapenems. In a different approach, CRISPR interference (CRISPRi) was used to lower the expression of the efflux pump AcrAB-TolC in *E. coli* and thereby increased the susceptibility to rifampicin, erythromycin and tetracycline^45^.

The successful application of CRISPR-Cas systems in elimination of AMR genes from bacterial populations suggest that it also can be efficient in preventing probiotic bacteria from acquiring AMR genes. In this study, we constructed a CRISPR-Cas9 system and evaluated its potential in protecting probiotic *E. coli* from acquiring AMR genes.

## Materials and methods

### Strains and growth conditions

All strains used in this study are derivatives of *Escherichia coli* K-12 MG1655 (Table S1) unless specified otherwise. All strains were grown in LB at 37 °C, shaking at 200 rpm unless specified otherwise. Where appropriate, liquid cultures and 1.5% Luria agar (LA) plates were supplemented with: Kanamycin (KAN) 50 mg/L, Chloramphenicol (CAM) 12.5 mg/L, Ampicillin (AMP) 100 mg/L, cefotaxime (CEFO) 10 mg/L, and diaminopimelic acid (DAP) 0.3mM.

### Strain constructions

CRISPR-Cas9 plasmid-carrying recipient strains were created by introducing either pWEB-TNC::pJ23104-*tracr*-pJ23101-*cas9* (hereafter denoted as pCRISPR^−^), or pWEB-TNC::pJ23104-*tracr*-pJ23101-*cas9-CRISPRsynt* (hereafter denoted as pCRISPR^+^) into MG1655. *tracr* under the control of BioBrick promoter pJ23104 was amplified from pCRISPathBrick using oligos SK1805 and SK2407 (Table S2) and cloned into pWEB-TNC using SalI and SmaI, while *cas9* under pJ23101 were amplified from p15a-cas9deg-amp using oligos SK1807 and SK2151 (Table S2) and cloned using SalI and BglII. In the final construct, *tracr* and *cas9* were cloned in opposite directions with or without the addition of leader and repeat-spacer array (Fig. S1) synthesized by Thermo Scientific. The constructs were verified using oligos SK1926-SK1928, SK1986, SK2030-SK2032, SK2419-SK2420.

Single gene targets were cloned into pBAD18-kan as previously described^46^ and were used as donor plasmids for transformation assays. Donor strains for transduction assays were constructed by amplifying the antibiotic resistance gene from pBAD18 together with the downstream *kan* gene found on the plasmid using oligos SK2414 and SK2415 (Table S2). The resulting PCR products were then integrated into the MG1655 chromosome downstream of the *lacA* gene by lambda red recombineering^47^. The *lacA* locus was selected as it has previously been shown to be neutral for incorporation of antibiotic markers^48^. Using Gibson Assembly^49^, donor plasmids for single target conjugation assays were constructed by introducing the target genes into pMM441-*kan*, a kanamycin-resistant variant of pMM441, and amplified using oligos SK2429 and SK2430 and verified with oligos SK2423-SK2426 (Table S2). These plasmids were then introduced into MG1655 with the *tra* genes from λ *pir* integrated into the *mhpC* locus on the chromosome^50^. Donor strains for conjugation of clinical plasmids were constructed by conjugation of resistance plasmids isolated from clinical *E. coli* and *K. pneumoniae* isolates into a MG1655 *dapA::cat* strain, which lacks the ability to produce di-aminopimelic acid (DAP).

## CRISPR-Cas9 efficiency assays

All efficiency assays were repeated at least 3 times. The exact number of biological replicates can be found in the figure legend of each experiment.

Transformation assays were performed by heat shock transformation of 100 ng of pBAD18::target donor plasmids into TSS competent^51^ recipient cells. The recipient cells carried a chloramphenicol resistance marker and the transferred plasmid carries a kanamycin resistance marker. After heat shock, cells were diluted and plated for viable counts on LB plates supplemented with CAM and CAM + KAN. The number of viable bacteria in each condition was determined by colony counting after ~ 16 h of incubation at 37 °C. From these numbers, the transformation efficiency was determined by dividing the number of transformants (CFU/ml on CAM + KAN plates) with the total number of recipient cells (CFU/ml on CAM plates). The data is presented as transformants per 10^9^ recipients.

Transduction assays were performed by infecting recipient cells with P1 phage lysates^52^ generated on *E. coli* MG1655 *lacA*::target donor strains. The recipient cells carried a chloramphenicol resistance marker and the phage lysates contained a kanamycin resistance gene in addition to the indicated target gene. The transduction efficiency was determined by comparing the number of transductants to the total number of recipient cells, scored on CAM + KAN and CAM plates, respectively as described for transformation above. Plates were also supplemented with 5 mM sodium citrate to limit super-infection by P1 phage^52^.

Conjugation assays were performed by mixing equal volumes of PBS-washed, 2x concentrated donor and recipient cells from overnight cultures. The conjugation mixes were then spotted on LA supplemented with AMP for pMM441-kan conjugations and LA plates for conjugation of clinical plasmids. The plates were incubated at 37 °C for 1 h for the pMM441-kan conjugations and 24 h for clinical plasmids. The recipient cells carried a chloramphenicol resistance marker and therefore the total number of recipient cells were scored by plating on LA supplemented with CAM, and successful transconjugants could be scored by plating on LA supplemented with CAM + KAN or CAM + CEFO for pMM441-kan (KAN) and clinical plasmids (CEFO), respectively. The conjugation efficiency is presented as transconjugants per 10^9^ recipients.

## Results

### Design of CRISPR spacers and construction of an antibiotic resistance gene-targeting CRISPR-Cas9 system

A prominent example of AMR spreading through HGT occurred at the Uppsala University Hospital in Sweden in 2005. A *Klebsiella pneumoniae* strain carrying the multi-drug resistance plasmid pUUH239.2 caused an outbreak with more than 300 patients affected^53^. During the outbreak, this resistance plasmid was found to transfer horizontally to the native microbiota within individual patients^54^. This highlights the significance of HGT as a driver of AMR^55^ and the risk that virulence or AMR genes could transfer to probiotic bacteria through HGT. In order to limit this risk, we constructed a CRISPR-Cas9 system aimed at preventing HGT of AMR genes to the cells that contain the system. CRISPR arrays contain a series of direct repeats separated by short spacers, which normally match previously encountered phage invaders. In our system, we designed the spacers to match eight of the 10 common AMR genes found on the pUUH239.2 plasmid; *mph(A)*,* dhfrXII*,* aadA2*,* sul1*, *bla*_OXA−1_, *bla*_CTX−M−15_, *aac(6´)-1b-cr* and *tetA* (*tetR*,* mrx* and *bla*_TEM−1_ were not included). The chosen genes are commonly encountered on large multiresistance plasmids in *Enterobacterales*. The *sul-1*,* dhfrXII*, and *aadA2* genes are located on a typical class 1 integron^56^, *mphAR-mrx* is the most common macrolide resistance determinant on clinical Gram negative plasmids^57^ and the *tetAR* variant is the most common tetracycline resistance determinant among human and animal *E. coli*^58,59^. The beta-lactamases TEM-1, OXA-1 and the extended spectrum beta-lactamase CTX-M-15 belong to the most frequent groups of beta-lactamase enzymes among resistant *E. coli* and especially CTX-M-15 is one of the most wide-spread and problematic ESBLs globally^60^. Taken together, these genes are relevant to study beyond the particular context of the Uppsala outbreak.

**Fig. 1:**
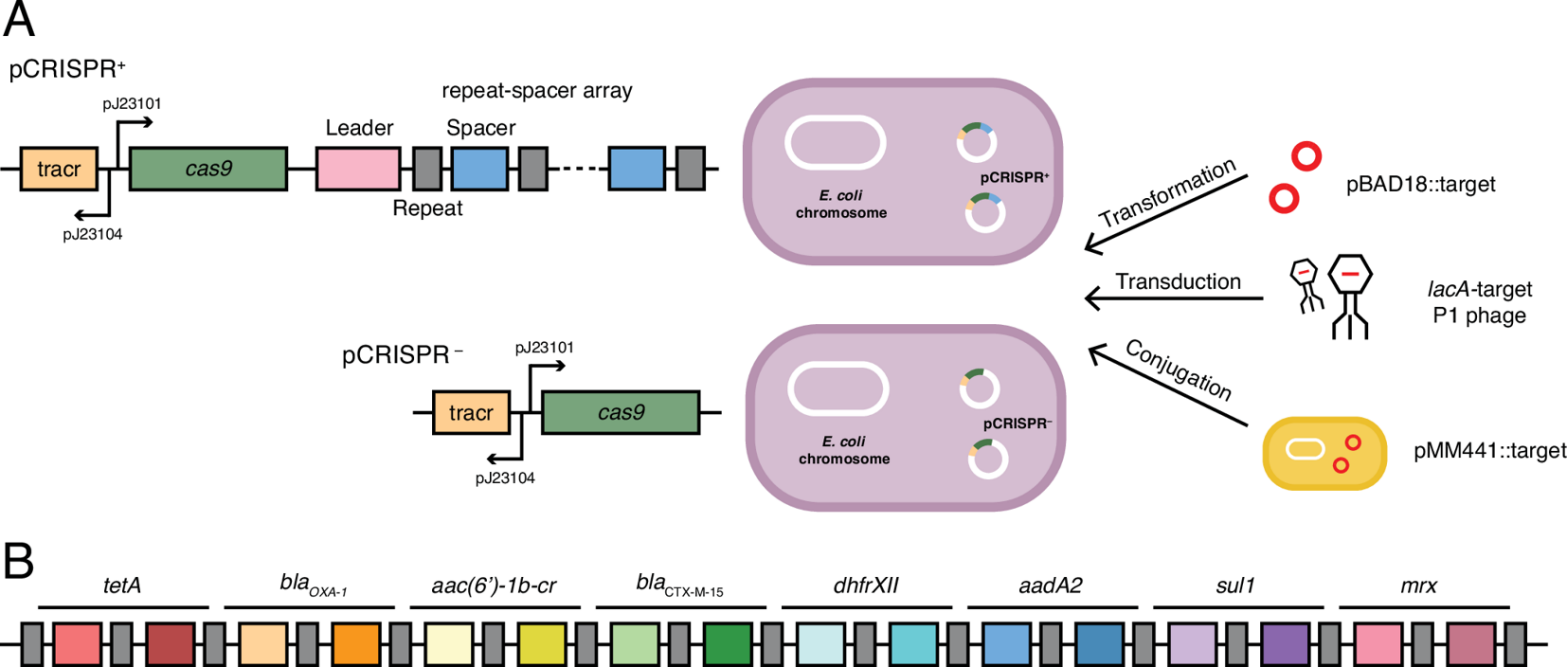
An antibiotic resistance gene-targeting CRISPR system. (**A**) The CRISPR array. Two spacers for each of the eight target resistance genes selected were incorporated into a CRISPR repeat-spacer array. Repeats are shown as grey boxes and spacers as coloured boxes. (**B**) *tracrRNA* and *cas9* are expressed from constitutive sigma_70_ promoters (J23101 and J23106). In addition, pCRISPR^+^ also carries a repeat-spacer array targeting eight different antibiotic resistance genes. pCRISPR^–^ lacks the repeat-spacer array. The efficiency of the system was tested using plasmid transformation, P1 phage transduction and conjugation.

In order to limit the transfer of these resistance genes, two spacer sequences per target gene were incorporated into the CRISPR array, resulting in a total of 16 spacers (Fig. 1A). As an effort to allow targeting of different gene variants, the spacer sequences were preferentially chosen from conserved regions of the genes. Conserved regions were identified by multiple sequence alignment of the different variants of the resistance genes found in the Bacterial Antimicrobial Resistance Reference Gene Database at NCBI (accession: PRJNA313047) and the selected spacers can be found in Fig. S1. The resulting CRISPR array was combined with trans-activating CRISPR *(tracr)* RNA and *cas9*, expressed from strong synthetic promoters, to generate a functional AMR gene-targeting CRISPR-Cas9 system (Fig. 1B). As a control, we also included a system expressing *tracrRNA* and *cas9* but lacking the CRISPR repeat-spacer array (Fig. 1B). We decided to use the CRISPR-Cas9 system as it requires a single RNA-guided DNA endonuclease^18^ and can be expressed heterologously in other organisms^61^, making it widely adaptable for genetic engineering purposes. For a first proof-of-principle, we constructed the system on a plasmid, which can easily be integrated onto the chromosome as shown previously^48^. To assess the CRISPR-Cas9 system, we introduced the constructs into *E. coli* K-12 MG1655.

### The CRISPR-Cas9 system protects against the acquisition of single gene-targets

**Fig. 2:**
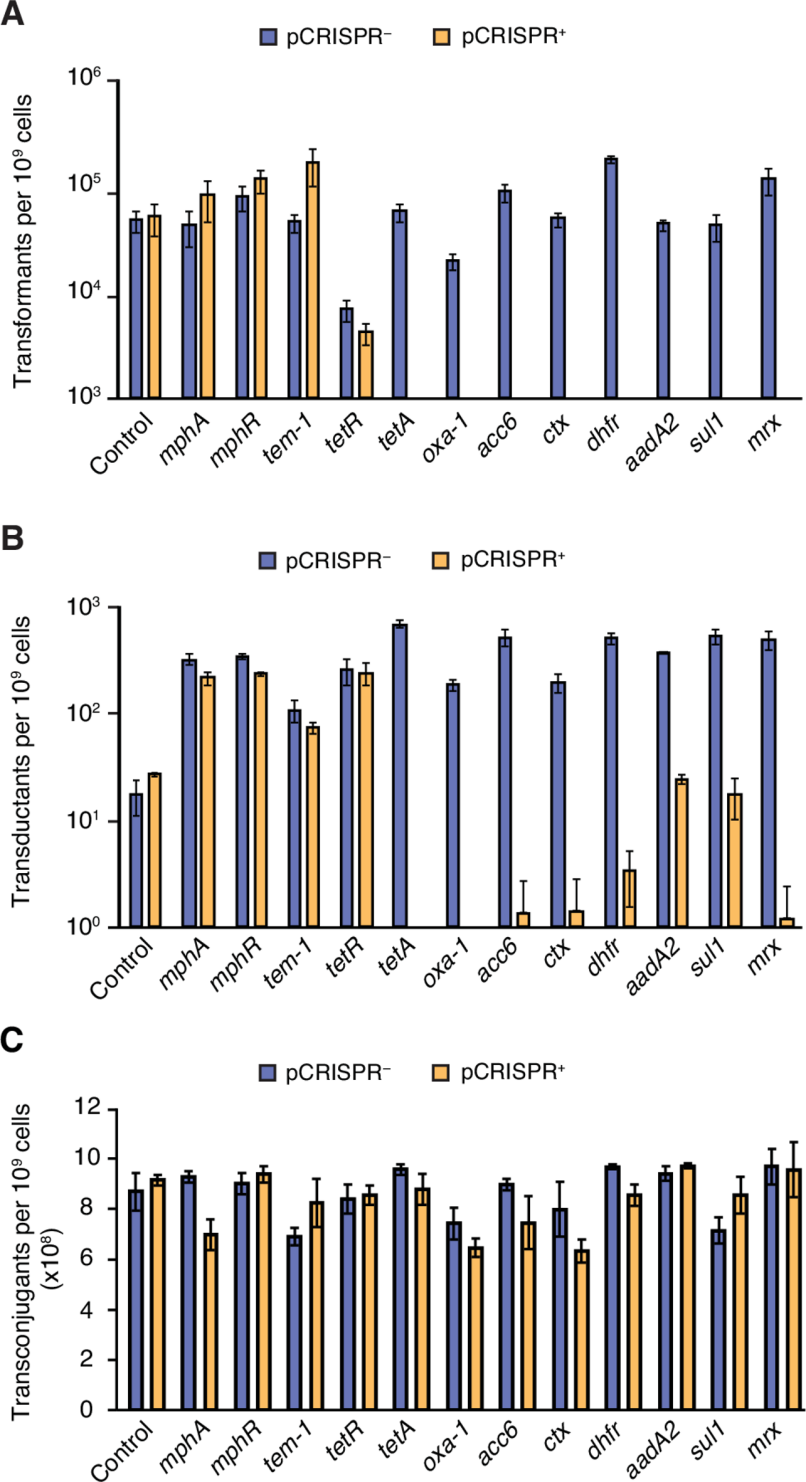
CRISPR system protection against single gene target acquisition in *E.* *coli* K-12 MG1655 without (pCRISPR^−^) or with (pCRISR^+^) the repeat-spacer array. Transfer through (**A**) transformation of pBAD18::target plasmids, (**B**) transduction of *lacA*::target by P1 phage and (**C**) conjugation of pMM441::target-kan plasmid. The CRISPR array carries spacers targeting *tetA(A)*, *bla*_OXA−1_, *aac(6´)-Ib-cr*, *bla*_CTX−M−15_, *dhfrXII*, *aadA2*, *sul1* and *mrx*. *n* = 3 biological replicates and error bars represent SEM.

To determine whether our CRISPR-Cas9 system can prevent HGT of AMR genes, we tested the system against single gene-target acquisition. First, we tested the CRISPR-Cas9 system against transformation of plasmids carrying one of the eight AMR genes targeted by the CRISPR array (Table 1). In addition, we also included four control genes originating from the pUUH239.2 plasmid, for which no matching spacers were included in the CRISPR array (Table 1). These twelve resistance genes (eight targeted and four controls) were cloned individually onto a pBAD-18 backbone. To test the efficacy of protection, we transformed the plasmids into competent *E. coli* K-12 MG1655 recipient strains carrying either the pCRISPR^-^ (missing the repeat-spacer array) or pCRISPR^+^ (having the repeat-spacer array) constructs. Equal amounts of the target plasmids were used for all transformations. We found that the CRISPR-Cas9 system effectively prevented the acquisition of plasmids carrying CRISPR-targeted AMR genes as no detectable transformants were observed for recipients armed with pCRISPR^+^ as compared to 10 000 to 100 000 transformants in the pCRISPR^-^ recipients (Fig. 2A). No reduction in control target acquisition was observed when comparing pCRISPR^+^ recipients with pCRISPR^-^ (Fig. 2A). Thus, the CRISPR-Cas9 system completely eliminated the acquisition of resistance genes through plasmid transformation.

**Table 1:** Single gene-targets tested against CRISPR-Cas9 protection. Four control genes missing matching spacers in the CRISPR array were used. The eight genes targeted by spacers in the CRISPR array are listed in the same order they are found in the array.

Target	Hereafter referred to as	Class of antibiotic resistance	Spacer in CRISPR array
Empty control	Control	–	–
*mphA*	*mphA*	Macrolide	–
*mphR*	*mphR*	Macrolide	–
*tem-1*	*tem-1*	Beta-lactam	–
*tetR*	*tetR*	Tetracycline	–
*tetA*	*tetA*	Tetracycline	+
*oxa-1*	*oxa-1*	Beta-lactam	+
*aac(6´)-1b-cr*	*aac6*	Aminoglycoside	+
*ctx-m-15*	*ctx*	Beta-lactam	+
*dhfrXII*	*dhfr*	Trimethoprim	+
*aadA2*	*aadA2*	Aminoglycoside	+
*sul1*	*sul1*	Sulphonamide	+
*mrx*	*mrx*	Macrolide	+

Next, we tested the CRISPR-Cas9 system against HGT mediated by phage. pCRISPR^-^/^+^ recipient cells were exposed to P1 phage lysates generated from donor strains with the AMR genes from Table 1, integrated on the chromosome downstream of the *lacA* gene. We scored the frequency of target gene integration (transduction frequency) by plating on CAM + KAN (positive transductants) vs. CAM plates (recipients). The CRISPR-Cas9 system limited the acquisition of the CRISPR-targeted genes by 1–3 logs (Fig. 2B). For some target genes, e.g. *oxa-1* and *tetA*, no detectable transductants could be observed in the pCRISPR^+^ recipients, whereas roughly 200 (*oxa-1*) and 700 (*tetA*) transductants were seen in the pCRISPR^-^ recipients. For other genes, e.g. *aadA2* and *sul1*, around 10 transductants per 10^9^ cells were obtained, but this was still 40–50 times lower than for pCRISPR^-^, where 400 (*aadA2*) and 500 (*sul1*) transductants were found. No non-specific reduction of control genes was observed (Fig. 2B), suggesting that the CRISPR-Cas9 system effectively limits HGT of the targeted genes mediated through transduction.

Acquisition of AMR genes through conjugative plasmids is a major cause of the spread of AMR among bacteria^62^. To test the efficacy of the CRISPR-Cas9 system against conjugation events, resistance genes were cloned into the highly efficient conjugative plasmid pMM441-kan. The observed conjugation frequencies ranged between 63 and 97% and were indistinguishable between pCRISPR^−^ and pCRISPR^+^ recipients for all target genes (Fig. 2C). These results suggest that the CRISPR-Cas9 system is unable to block HGT mediated by conjugation under these conditions. Overall, our findings suggest that this CRISPR-Cas9 system may have a limit of reducing target gene acquisition by 1–3 logs through HGT by transformation and transduction but fails to block the transfer of the highly conjugative plasmid pMM441.

### The CRISPR-Cas9 system limits the transfer of clinical plasmids

**Fig. 3:**
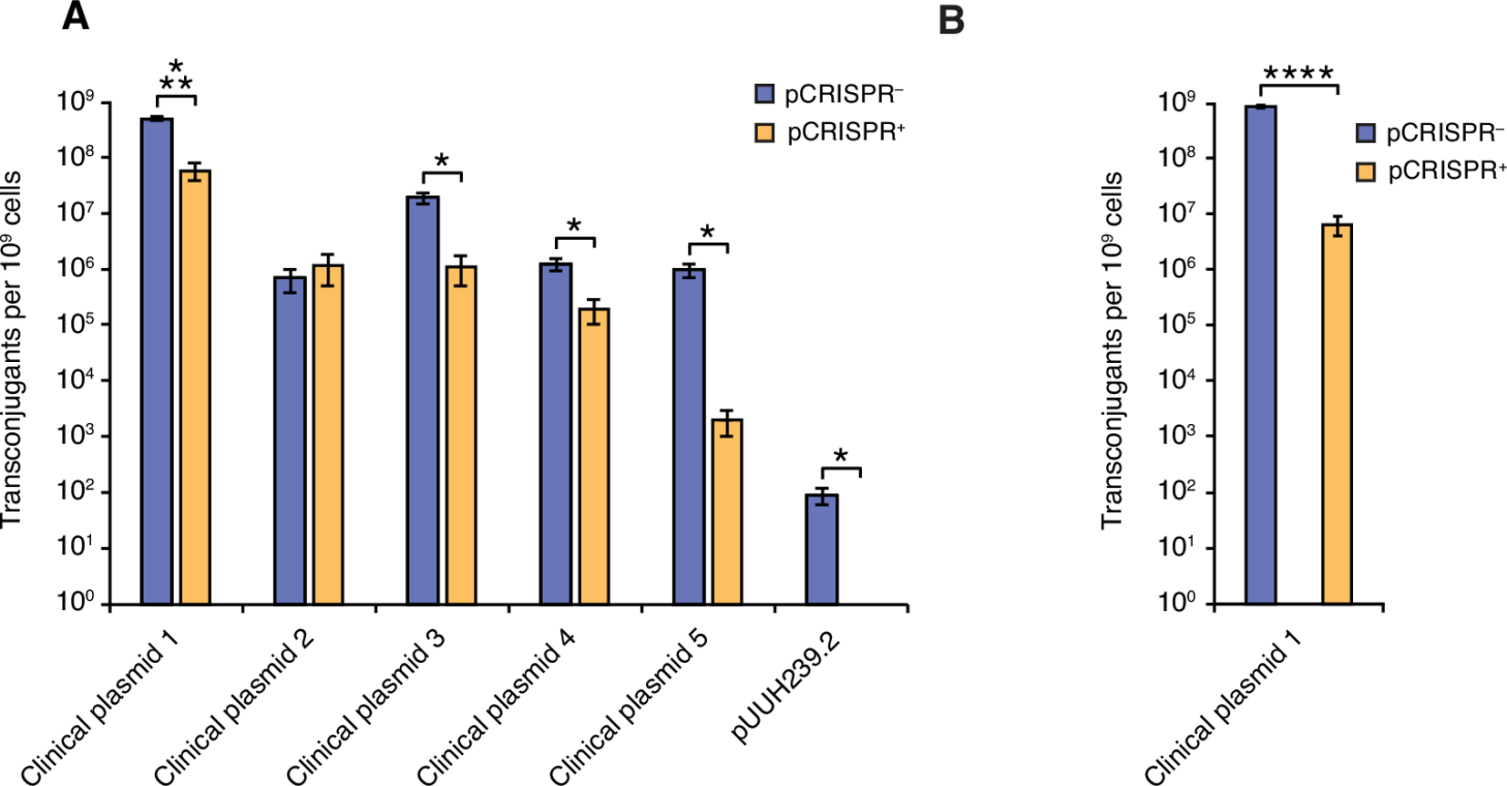
The CRISPR system limits HGT through conjugation of clinical plasmids. (**A**) Conjugation of five clinical plasmids encoding *bla*_CTX−M−15_ and the pUUH239.2 plasmid into *E.* *coli* K-12 MG1655 without (pCRISPR^−^) or with (pCRISR^+^) the repeat-spacer array. (**B**) Conjugation of the clinical plasmid 1 encoding *bla*_CTX−M−15_ into the probiotic bacterium *E.* *coli* Nissle 1917 equipped with or without the CRISPR array. *n* = 5 biological replicates and error bars represent the SEM.

The CRISPR-Cas9 system was unable to reduce the conjugative uptake of pMM441 encoding the single AMR genes. However, the conjugation frequency in this assay was very high since the pMM441-plasmid has a constitutive expression of the conjugation machinery, in contrast to most natural plasmids. Thus, the lack of CRISPR-mediated protection could result from the artificially high conjugation rate. To investigate this further, we tested the protective ability of the CRISPR-Cas9 system against the acquisition of natural plasmids through conjugation. We used a set of clinical plasmids with one or several resistance genes targeted by our CRISPR-Cas9 system, including pUUH239.2 with eight targeted genes (Table 2). The presence of the CRISPR-Cas9 system significantly reduced the conjugation frequency of four out of five clinical plasmids by 1–3 log, depending on the plasmid (Fig. 3A). No protection could be seen for clinical plasmid 2. This is expected as the CRISPR-Cas9 system lacks spacers against this plasmid (Table 2). Interestingly, the CRISPR-Cas9 system effectively blocked the very high conjugation frequency of clinical plasmid 1, even though this frequency was similar to pMM441 where no protection could be seen. In addition, the system effectively eliminated the transfer of pUUH239.2 with no transconjugants observed in any of our conjugation attempts (Fig. 3A). The CRISPR-Cas9 system has 16 spacers against both clinical plasmid 1 and pUUH239.2 as compared to the two spacers seen for pMM441 and clinical plasmids 4 and 5. Thus, our results suggest that by increasing the number of spacers protection can be increased also in the presence of very high conjugation frequencies.

**Table 2:** Clinical plasmids used for conjugation, their size, type, and resistance genes. Genes targeted by the CRISPR-Cas9 system are highlighted in bold. Total number of spacers targeting the plasmid is given in bold (no of spacer).

Plasmid number	Plasmid	Size	InC	Resistance genes	No of spacers	Refs.
Clinical plasmid 1	p4/1-1.1	181 kbp	FIA, FIB, FII	*aph(3’)-1a*, ***bla***_***CTX−M−15***_, ***sul1***, *bla*_*TEM−1b*_, *mph(A)*, ***tetA***, *catB3 (partial)*, ***bla***_***OXA−1***_, ***aac(6’)-Ib-cr***, ***aadA2***, ***dfrA12***, ***mrx***	**16**	^63^
Clinical plasmid 2	pKF3-70-like	70 kbp	FII	*bla*_*CTX−M−14*_	**0**	^64^
Clinical plasmid 3	Unnamed	67 kbp	F	***aac(6’)-1b-cr***, *aac(3)-IId*,* aadA5*,* aph(3’’)-1b*,* aph(6)-1d*,* bla*_*CTX−M−14*_, *cm1*,* drfA17*,* mph(A)*, ***sul2***, ***tetA***,*** mrx***	**7**	^64^
Clinical plasmid 4	Unnamed	121 kbp	I	*aph(3’)-1a*,* aph(3’’)-1b*,* aph(6)-1d*,* bla*_*CTX−M−65*_, *bla*_*TEM−104*,_*bla*_*TEM−1B*_, *bla*_*TEM−198*_, *bla*_*TEM−234*_, *catA1*,* fosA3*,* sul2*, ***tetA***	**2**	^64^
Clinical plasmid 5	Unnamed	55 kbp	unknown	***bla***_***CTX−M−1***_	**2**	^64^
pUUH239.2	pUUH239.2	220 kbp	IncFII_K_	*mph(A)*, ***mrx***, ***sul1***, ***aadA2***, ***dhfrXII***, *bla*_TEM−1_, ***bla***_**CTX−M−15**_, ***bla***_**OXA−1**_, ***aac(6´)-1b-cr***, ***tetA***	**16**	^64^

### The CRISPR-Cas9 system prevents conjugation of a clinical plasmid into the probiotic bacterium *E.coli* Nissle 1917

The CRISPR-Cas9 system provided protection against HGT in an *E. coli* laboratory strain. To determine the efficiency of the CRISPR-Cas9 system in a known probiotic strain, we introduced the pCRISPR^+^ vector and the negative control pCRISPR^-^, lacking the CRISPR array, to the well-characterized probiotic *E. coli* strain Nissle 1917. Using the clinical plasmid with the highest conjugation frequency, clinical plasmid 1, we demonstrated that the presence of the CRISPR-Cas9 system protects the probiotic *E. coli* Nissle 1917 from HGT through conjugation by a roughly 100-fold reduction in efficiency (Fig. 3B). This is approximately 10 times more than what was observed with *E. coli* MG1655, where we saw a 10-fold reduction. These results suggest that our CRISPR-Cas9 system can effectively be used to defend bacteria against the acquisition of antibiotic resistance plasmids.

## Discussion

Our CRISPR-Cas9 system has demonstrated promising results in limiting the acquisition of AMR genes transferred through transformation, transduction, as well as conjugation. This could be very useful in protecting probiotic bacteria from becoming superbugs through HGT events. Transfer of genetic elements that encode virulence factors can have adverse consequences if taken up by non-pathogenic probiotic strains^65^, limiting the safe use of live probiotics. With recent advances in bacteria-based drug delivery for diseases such as inflammatory bowel diseases (reviewed in^66^), the need for safe probiotics will also grow. In our system, the CRISPR spacers can be customized to target any gene of interest, including genes encoding virulence factors such as toxins. The modular design of the system, makes its components easily exchangeable, to the desired application. In addition to replacing the spacers, the expression level of Cas9 can be fine-tuned by introducing constitutive promoters of different strengths or promoters that are inducible in situ^67^ or by fusing degradation tags to the Cas9 protein to promote faster turnover of the protein^68^. The system can also be adapted to safeguard other groups of bacteria against HGT, e.g. to prevent hydrocarbon-degrading bacteria used in oil clean-up^69^ from becoming virulent or to protect bacteria used in industrial fermentation from phage infections^70^. Although we chose a plasmid-based approach for a first proof-of-principle, the plasmid-based system can easily be integrated onto the chromosome^48^. Chromosomal integration is important both for stable maintenance of the system in the probiotic strain (i.e. reducing the risk of loss) as well for limiting spread of the system to other unwanted bacteria. Here we limited our study to *E. coli*, but the system could easily be modified for use in other probiotic bacteria including engineered *Lactobacilli.*

Numerous attempts to utilize CRISPR technology in treatment of bacterial infections have been performed since the discovery of the system^36–43^. Recent attempts have successfully manage to use CRISPR to decrease the level of specific bacteria in the normal microbiota of mice^71^ and to genetically manipulate specific strains in the host^72^. To our knowledge, fewer publications have focused on trying to protect strains from HGT^34^. Our CRISPR-Cas9 system was initially designed to protect probiotic bacteria from acquiring genes, but the system could also be delivered using lambdoid phages as the systems is located on a large plasmid containing a Cos-site^73^. Future attempts could be directed to compare the efficacy of this system to the evolved Cas9-base-modification systems tested in^72,74^, or the type-I-E CRISPR system used in^71^.

CRISPR spacers are commonly found to protect against HGT of plasmids as well as phage in nature^33,34^, suggesting that a protective approach could be functional. However, we found that although the presence of CRISPR spacers effectively reduced the acquisition of AMR genes through HGT, it was insufficient to protect against the uptake of plasmids with high conjugation efficiencies (Fig. 2C), when only two spacers targeted the plasmid. Protection could be achieved when more spacers targeted the conjugative plasmid, also at very high conjugation efficiency. These very high rates of conjugation are rather unlikely to occur in nature, but it is important to note that the system does not provide 100% protection on its own. It is also worth noting that if used alone, resistance against this artificial HGT prevention system will likely evolve. E.g. anti-CRISPR systems naturally found in phage could render the system less effective in long-term applications^75^ or escape mutants or phage with increased replication rates could avoid or overwhelm CRISPR surveillance^76^. Future attempts should focus on making the system more efficient by e.g. increasing expression of Cas9 or by including more spacers against a single target. The latter could help also against escape mutants as previous reports suggest that CRISPR evasion decreases when multiple and diverse spacers target the DNA of interest^77^.

In conclusion, our CRISPR-Cas9 system can be valuable as one of several defence mechanisms that can be employed together to further reduce the risk of unwanted gene acquisitions in GMM probiotics, including biocontained strains where the system could be wired to prevent escape of the containment through HGT. A specific example of how CRISPR could be used for optimizing the safety of probiotics comes from the recent finding that *E. coli* Nissle 1917, similarly to other *E. coli* of the B2 phylogenetic group, contains the *pks* island encoding for the DNA damaging toxin colibactin^78^. Colibactin is known to cause DNA breaks in intestinal epithelial cells of the gut in gnotobiotic mice^79^. This toxicity can be alleviated by a mutation in the colibactin encoding *clbP* gene^79^, but such a mutation could easily be reverted by a HGT event from any closely related *E. coli* strain from the B2 phylogenetic group, making the safety of using *E. coli* Nissle in probiotic applications questionable. Thus, in order to use genetically engineered *E. coli* Nissle as a safe probiotic, a biocontainment strategy that prevents HGT as the one described here, could be useful.

## Electronic supplementary material

Below is the link to the electronic supplementary material.

Supplementary Material 1

Supplementary Table S3

## Data Availability

All raw data for the manuscript is available as a supplementary excel file (Table S3).
