# Supplementary material for: A CRISPR-Cas9 system protecting E. coli against acquisition of antibiotic resistance genes
Source: Sci Rep. 2025 Jan 9;15:1545. doi: 10.1038/s41598-025-85334-2 (PMC11718013; doi:10.1038/s41598-025-85334-2)
Supplement: Supplementary file 1 — Supplementary Material 1 [file 41598_2025_85334_MOESM1_ESM.docx]

**Supplementary material for: A CRISPR system protecting *E. coli* against acquisition of antibiotic resistance genes**

Danna Lee^1†^, Petra Muir^1†^, Sara Lundberg^1^, August Lundholm^1^, Linus Sandegren^2*^ and Sanna Koskiniemi^1*^

**This file includes:**

**Supplementary figure 1**

**Supplementary tables 1-2**

**Legend for supplementary table 3**

**Supplementary references**

**Figure S1. The CRISPR repeat-spacer array.** Overview of the synthetic CRISPR array. The crRNA leader is marked in blue and spacers targeting different resistance genes are marked in red.

**Table S1. Bacterial strain used in this study.**

| Strain number | Genotype | Origin |
| --- | --- | --- |
| SK3524 | Eco MG1655 *DELdap* /pUUH239.2 | [1] |
| SK5384 | Eco MG1655 *lacIZYA::FRT, galK::sYFP2-bla, dapA::cat, rifR* /plasmid#1 | [2] |
| SK5386 | Eco MG1655 *lacIZYA*::FRT, *galK::sYFP2-bla*, *dapA::cat*, rifR /plasmid#2 | [3] |
| SK5387 | Eco MG1655 *lacIZYA::FRT, galK::sYFP2-bla, dapA::cat, rifR* /plasmid#3 | [3] |
| SK5388 | Eco MG1655 *lacIZYA::FRT, galK::sYFP2-bla, dapA::cat, rifR* /plasmid#4 | [3] |
| SK5389 | Eco MG1655 *lacIZYA::FRT, galK::sYFP2-bla, dapA::cat, rifR* /plasmid#5 | [3] |
| SK5465 | Eco MG1655 /pBAD18 | [4] |
| SK5466 | Eco MG1655 /pBAD18::*bla(CTX-M-15)* | [4] |
| SK5467 | Eco MG1655 /pBAD18::*bla(TEM-1)* | [4] |
| SK5468 | Eco MG1655 /pBAD18::*bla(OXA-1)* | [4] |
| SK5469 | Eco MG1655 /pBAD18::*mphA* | [4] |
| SK5470 | Eco MG1655 /pBAD18::*mrx* | [4] |
| SK5471 | Eco MG1655 /pBAD18::*mphR* | [4] |
| SK5472 | Eco MG1655 /pBAD18::*aac(6')-Ib-cr* | [4] |
| SK5473 | Eco MG1655 /pBAD18::*sul-1* | [4] |
| SK5474 | Eco MG1655 /pBAD18::*aadA2* | [4] |
| SK5475 | Eco MG1655 /pBAD18::*dhfr* | [4] |
| SK5476 | Eco MG1655 /pBAD18::*tetR* | [4] |
| SK5477 | Eco MG1655 /pBAD18::*tetA* | [4] |
| SK5758 | Eco MG1655 *ds-lacA::kan* | This study |
| SK5759 | Eco MG1655 *ds-lacA::kan-ctx-m-15* | This study |
| SK5760 | Eco MG1655 *ds-lacA::kan-tem1* | This study |
| SK5761 | Eco MG1655 *ds-lacA::kan-oxa1* | This study |
| SK5762 | Eco MG1655 *ds-lacA::kan-mphA* | This study |
| SK5763 | Eco MG1655 *ds-lacA::kan-mrx* | This study |
| SK5764 | Eco MG1655 *ds-lacA::kan-mphR* | This study |
| SK5765 | Eco MG1655 *ds-lacA::kan-aac6* | This study |
| SK5766 | Eco MG1655 *ds-lacA::kan-sul1* | This study |
| SK5767 | Eco MG1655 *ds-lacA::kan-aadA2* | This study |
| SK5768 | Eco MG1655 *ds-lacA::kan-dhfr* | This study |
| SK5769 | Eco MG1655 *ds-lacA::kan-tetR* | This study |
| SK5770 | Eco MG1655 *ds-lacA::kan-tetA* | This study |
| SK5771 | Eco MG1655 / pWEB-TNC::pJ23104-*tracr*-pJ23101-*cas9* | This study |
| SK5772 | Eco MG1655 / pWEB-TNC::pJ23104-*tracr*-pJ23101-*cas9*-*CRISPRsynt* | This study |
| SK5775 | Eco Nissle 1917 / pWEB-TNC::pJ23104-*tracr*-pJ23101-*cas9* | This study |
| SK5776 | Eco Nissle 1917 / pWEB-TNC::pJ23104-*tracr*-pJ23101-*cas9*-*CRISPRsynt* | This study |
| SK5946 | Eco MG1655 *rph+* RP4-2-Tc::[*ΔMu1::aac(3)IV-ΔaphA-Δnic35-ΔMu2::zeo*] / pMM441-*kan* | This study |
| SK5947 | Eco MG1655 *rph+* RP4-2-Tc::[*ΔMu1::aac(3)IV-ΔaphA-Δnic35-ΔMu2::zeo*] / pMM441-*kan::ctx-m-15* | This study |
| SK5948 | Eco MG1655 *rph+* RP4-2-Tc::[*ΔMu1::aac(3)IV-ΔaphA-Δnic35-ΔMu2::zeo*] / pMM441*-kan::tem1* | This study |
| SK5949 | Eco MG1655 *rph+* RP4-2-Tc::[*ΔMu1::aac(3)IV-ΔaphA-Δnic35-ΔMu2::zeo*] / pMM441*-kan::oxa1* | This study |
| SK5950 | Eco MG1655 *rph+* RP4-2-Tc::[*ΔMu1::aac(3)IV-ΔaphA-Δnic35-ΔMu2::zeo*] / pMM441-*kan::mphA* | This study |
| SK5951 | Eco MG1655 *rph+* RP4-2-Tc::[*ΔMu1::aac(3)IV-ΔaphA-Δnic35-ΔMu2::zeo*] / pMM441-*kan::mrx* | This study |
| SK5952 | Eco MG1655 *rph+* RP4-2-Tc::[*ΔMu1::aac(3)IV-ΔaphA-Δnic35-ΔMu2::zeo*] / pMM441-*kan::mphR* | This study |
| SK5953 | Eco MG1655 *rph+* RP4-2-Tc::[*ΔMu1::aac(3)IV-ΔaphA-Δnic35-ΔMu2::zeo*] / pMM441-*kan::aac6* | This study |
| SK5954 | Eco MG1655 *rph+* RP4-2-Tc::[*ΔMu1::aac(3)IV-ΔaphA-Δnic35-ΔMu2::zeo*] / pMM441-*kan::sul1* | This study |
| SK5955 | Eco MG1655 *rph+* RP4-2-Tc::[*ΔMu1::aac(3)IV-ΔaphA-Δnic35-ΔMu2::zeo*] / pMM441-*kan::aadA2* | This study |
| SK5956 | Eco MG1655 *rph+* RP4-2-Tc::[*ΔMu1::aac(3)IV-ΔaphA-Δnic35-ΔMu2::zeo*] / pMM441-*kan::dhfr* | This study |
| SK5957 | Eco MG1655 *rph+* RP4-2-Tc::[*ΔMu1::aac(3)IV-ΔaphA-Δnic35-ΔMu2::zeo*] / pMM441-*kan::tetR* | This study |
| SK5958 | Eco MG1655 *rph+* RP4-2-Tc::[*ΔMu1::aac(3)IV-ΔaphA-Δnic35-ΔMu2::zeo*] / pMM441*-kan::tetA* | This study |

**Table S2. Oligos used in this study.**

| **Oligo** | **Description** | **Sequence (5’ to 3’)** |
| --- | --- | --- |
| SK1805 | tracer_F (smaI) | GCGCCCCGGGTTACGAAATCATCCTGTGG |
| SK1807 | Cas9_F (salI) | ATATGTCGACTTTACAGCTAGCTCAGTCCTA  GGTATTATGCTAGCGCAATTAGAGGGCTCAA  TGG |
| SK1926 | pWEB-TNC_F | GTGCCACCTGACGTCTAAG |
| SK1927 | pWEB-TNC_R | CATACACGGTGCCTGACTG |
| SK1928 | cas9_verF | CAGCTTAAACGTCGCCGTTA |
| SK1986 | spacer_verF | CGAATCTTGGAGCTCCCGC |
| SK2030 | Cas9 seq - F 1 | CTGATAAAGCGGATTTGCGC |
| SK2031 | Cas9 seq - F 2 | GTGAAGATTTGCTGCGCAAG |
| SK2032 | Cas9 seq - F 3 | TTGGAGACAACTTCTAAACGCC |
| SK2151 | Cas9 - detag – bglII_R | GCGCAGATCTTCTAGACTCGAGTTAGTCACCTC  CTAGCTGACTCAAATC |
| SK2407 | PJ23104 tracr (SalI)_F | ATATGTCGACTTGACAGCTAGCTCAGTCCTAGG  TATTGTGCTAGCTTGTTGGAACCATTCAAAACA  GCATAGC |
| SK2414 | ds-lacA::pBAD18_F | ATTATAAAAATTGCCTGATACGCTGCGCTTATC  AGGCCTAGGCGTCACACTTTGCTATGCC |
| SK2415 | ds-lacA::pBAD18_R | TGTTCATGCCGGATGCGGCTAATGTAGATCGCT  GAACTTGATGCTCTGCCAGTGTTACAACC |
| SK2419 | PJ23104 screen long_F | TTGACAGCTAGCTCAGTCCTAGGTATTGTGCTA  GC |
| SK2420 | cas9 screening_R | CATCCGACGCTATTTGTGCCG |
| SK2423 | pMM441 screening_F | GTCTCATTTTCGCCAGATATCGACG |
| SK2424 | pMM441 screening_R | GGGGACAGCAAGCGAACC |
| SK2425 | pMM441 ab screen_R | GGCAACCGAGCGTTCTGAAC |
| SK2426 | pMM441 ab screen_F | GATCGGCACGTAAGAGGTTCC |
| SK2429 | pMM441::target GA_F | TAGGCGTATCACGAGGCCCTTTCGTCTTCACCC  TGCTCCATACCCGTTTTTTTGGGCTAG |
| SK2430 | pMM441::target GA_R | GGCCCCGCCCAAAAAAATAGATCCTTAATCAT  CATCTCTCATCCGCCAAAACAGCC |

**Table S3. Raw data.**

Raw data for figures 1-3 is found as a separate excel file.

**References:**

1. Lytsy, B., et al., *The first major extended-spectrum beta-lactamase outbreak in Scandinavia was caused by clonal spread of a multiresistant Klebsiella pneumoniae producing CTX-M-15.* APMIS, 2008. **116**(4): p. 302-8.

2. Brolund, A., et al., *Dynamics of Resistance Plasmids in Extended-Spectrum-beta-Lactamase-Producing Enterobacteriaceae during Postinfection Colonization.* Antimicrob Agents Chemother, 2019. **63**(4).

3. Brolund, A., et al., *Plasmidome-analysis of ESBL-producing escherichia coli using conventional typing and high-throughput sequencing.* PLoS One, 2013. **8**(6): p. e65793.

4. Rajer, F., et al., *Evolutionary Trajectories toward High-Level beta-Lactam/beta-Lactamase Inhibitor Resistance in the Presence of Multiple beta-Lactamases.* Antimicrob Agents Chemother, 2022. **66**(6): p. e0029022.
